# The growing importance of genetics in human reproduction and development

**DOI:** 10.3325/cmj.2023.64.305

**Published:** 2023-10

**Authors:** Davor Ježek

**Affiliations:** 1 Department of Histology and Embryology, University of Zagreb, School of Medicine, Zagreb, Croatia; 2 Department for Transfusion Medicine and Transplantation Biology, Zagreb University Hospital, Zagreb, Croatia; 3 Scientific Centre of Excellence for Reproductive and Regenerative Medicine, University of Zagreb, School of Medicine, Zagreb, Croatia; *davor.jezek@mef.hr*

Human reproduction and growth depend on normal developmental processes such as the production of competent gametes, fertilization, embryonic development, pregnancy, and birth. The characteristics of a newborn are determined by specific genes on maternally and paternally inherited chromosomes. Forty-six chromosomes in humans bear approximately 23 000 genes. If genes are located on the same chromosome, they are usually inherited together and are known as linked genes. Human fertility or infertility is determined by several biological processes, including organ formation and development, neuroendocrine regulation, hormone production, meiosis, and mitosis (1-3).

If one takes the example of neuroendocrine regulation, under normal physiological conditions, a precise arrangement of hypothalamic kisspeptin neurons and gonadotropin-releasing hormone neurons induces the secretion of follicle-stimulating hormone (FSH) and luteinizing hormone (LH) from the anterior pituitary. Adequate secretion of gonadotropins initiates gonadal steroid production, which regulates testicular or ovarian development. Hormones produced by the testes or ovaries then exert feedback effects on the hypothalamus and pituitary to adjust the concentrations of FSH and LH. Defects at one or more levels of the hypothalamic-pituitary-gonadal axis can cause hypogonadism, which manifests as a reduction in sex hormones, absent or delayed puberty, and abnormal function of the gonads. Hypogonadotropic hypogonadism refers to an insufficient release of gonadotropin-releasing hormone or gonadotropin caused by dysfunction of the pituitary gland or the hypothalamus. More than 40 genes have been associated with the pathogenesis of congenital hypogonadotropic hypogonadism (4,5).

Chromosomal abnormalities, which can be numerical or structural, are frequent causes of birth defects and spontaneous abortions. Even in healthy young couples, 50% of conceptions end in a spontaneous abortion, and 50% of these have significant chromosomal abnormalities. The most common chromosomal abnormalities are Turner syndrome, triploidy, and trisomy 16. Chromosomal abnormalities account for 10% of major birth defects, and gene mutations account for an additional 8%. Microdeletions spanning only a few adjoining genes may result in a microdeletion or contiguous gene syndrome. Identifying genetic pathways controlling gamete and somatic cell differentiation is vital for future diagnostics, especially concerning azoospermia. Owing to emerging genetic panels, patients could be spared from unnecessary conventional or microtesticular sperm extraction procedures in the near future. In addition, novel marker genes identified in the frame of single-cell sequencing (scRNA-seq) studies may be included in genetic screening in the expanding field of reproductive genetics. This could lead to new infertility treatments, including stem cell differentiation, *in vitro* spermatogenesis/ovary, and gene therapy (6,7).

Several articles in this issue of the *Croatian Medical Journal* indicate the importance of genetic diagnosis and screening. The article by Sansović et al (8) highlights the importance of clinical evaluation in subjects with familial hearing loss and the research on family segregation in assessing the pathogenicity of variants in the α-tectorin gene. Odak et al (9) focused on the role of the *KMT5B* gene in the pathogenesis of neurodevelopmental disorders. The *KMT5B* gene, discovered a few years ago, is related to abnormal activities of enzymes that regulate histone activity and gene expression during brain development. A comprehensive clinical and molecular approach to patients with KMT5B variants is necessary to optimize their treatment. Another case report, authored by Morožin-Pohovski et al (10), describes a boy with microcephaly, trigonocephaly, and dysmorphic features. This is very likely the first report in the literature describing a patient who inherited 16p11.2 duplications from both parents. The article by Fučić et al (11) deals with the early environmental exposure of the mother and the child to pesticides. The authors found that interleukin-2 levels, hypomethylation of interleukin-2 gene site 1, and the mother’s rural residence, probably due to pesticide exposure, were predictive biomarkers for preterm birth. Finally, the article by Hat et al (12) focused on aging and sexual dimorphism of the human lacrimal gland.

The diagnostic and therapeutic procedures in human reproduction and development will soon include more advanced genetic testing, multi-omics analysis, microbiota treatment, germline nuclear transfer, *in vitro* gametogenesis, gene therapy, and increased application of artificial intelligence.
